# Mycobacterium tuberculosis Transmission between Cluster Members with Similar Fingerprint Patterns

**DOI:** 10.3201/eid0811.020284

**Published:** 2002-11

**Authors:** Kashef Ijaz, Zhenhua Yang, H. Stewart Matthews, Joseph H. Bates, M. Donald Cave

**Affiliations:** * Centers for Disease Control and Prevention, Atlanta, Georgia, USA; † University of Michigan, Ann Arbor, Michigan, USA; ‡ Arkansas Department of Health, Little Rock, Arkansas, USA; § University of Arkansas for Medical Sciences, Little Rock, Arkansas, USA; ¶ Central Arkansas Veterans Healthcare Services, Little Rock, Arkansas, USA

**Keywords:** *Mycobacterium tuberculosis*, IS*6110* RFLP, molecular epidemiology, tuberculosis transmission, bars

## Abstract

Molecular epidemiologic studies provide evidence of transmission of *Mycobacterium tuberculosis* within clusters of patients whose isolates share identical IS*6110*-DNA fingerprint patterns. However, *M. tuberculosis* transmission among patients whose isolates have similar but not identical DNA fingerprint patterns (i.e., differing by a single band) has not been well documented. We used DNA fingerprinting, combined with conventional epidemiology, to show unsuspected patterns of tuberculosis transmission associated with three public bars in the same city. Among clustered TB cases, DNA fingerprinting analysis of isolates with similar and identical fingerprints helped us discover epidemiologic links missed during routine tuberculosis contact investigations.

The use of DNA fingerprinting has led to important advances in understanding the epidemiology of tuberculosis (TB) (*1*–*5*). However, *Mycobacterium tuberculosis* isolates that possess fewer than six copies of IS*6110* do not generate sufficient polymorphism to be readily distinguishable by this technique and therefore require secondary genotyping with another probe (*6*). A few strains of *M. tuberculosis* lack IS*6110* and cannot be fingerprinted with this technique (*7*,*8*). Although the epidemiologic importance of *M. tuberculosis* strains that have more than five copies of IS*6110* with similar but not identical fingerprint patterns (differing by one or two IS*6110* hybridizing bands) is unknown, in some cases epidemiologic links among patients infected with such strains have been established (*9*). Failure to identify these similar, but not identical, strains results in a misinterpretation of the extent of TB transmission in a community.

We conducted this study to determine the epidemiologic evidence of transmission among patients whose isolates have similar DNA fingerprint patterns (i.e., differing by a single band). Another purpose was to evaluate how useful such evidence, when combined with conventional epidemiology, would be in determining epidemiologic links that may have been missed during routine TB contact investigations in a cluster of patients.

## The Study

We obtained *M. tuberculosis* isolates from persons whose cultures were positive for TB from the Arkansas Department of Health and from hospital and reference laboratories where clinical samples from Arkansas patients were submitted. At least one isolate was obtained for each person who had TB. During the period of study (January 1992–December 2000), we identified 1,977 patients with TB; 1,495 were culture positive. IS*6110*-based restriction fragment length polymorphism (RFLP) analysis on the DNA extracted from the isolates of 1,141 patients (77% of culture-positive patients) was performed as described previously (*10*). Identical DNA fingerprint patterns are present when two or more patients’ isolates have indistinguishable IS*6110*-DNA fingerprints of six or more bands or when isolates with fewer than six copies of IS*6110* have identical RFLP patterns and secondary typing with polymorphic GC-rich sequence (PGRS) shows them to be indistinguishable (*10*–*13*). Similar DNA fingerprints exist when two or more patients’ isolates share an IS*6110*-DNA fingerprint that differs by a single band (i.e., has an additional band [+ 1], lacks a band [- 1], or differs in the size of a single hybridizing band) and has an identical pattern by PGRS (*10*–*13*). We performed secondary typing with PGRS for identical IS*6110*-based DNA fingerprint patterns with fewer than six bands or for patterns with six or more bands that were similar but differed by a single band (*10*–*13*).

When DNA fingerprints for isolates analyzed from 1992 to 2000 were reviewed for clustering, we identified a cluster of 15 TB patients. For all patients in the cluster, we examined routine TB contact investigation records, medical histories, and laboratory records. By using standard interviews, we established epidemiologic links among case-patients prospectively and retrospectively.

Eleven patients in the cluster resided in a small city in a predominantly rural county (county 1) with a population of 80,268 (*14*). The other four patients with isolates showing this DNA fingerprint resided in three other Arkansas counties, including one geographically contiguous to county 1. The other two counties were in different parts of the state and were not geographically contiguous to each other. Patients with isolates displaying this DNA fingerprint were not encountered at any other site during the study period.

**Figure 1:**
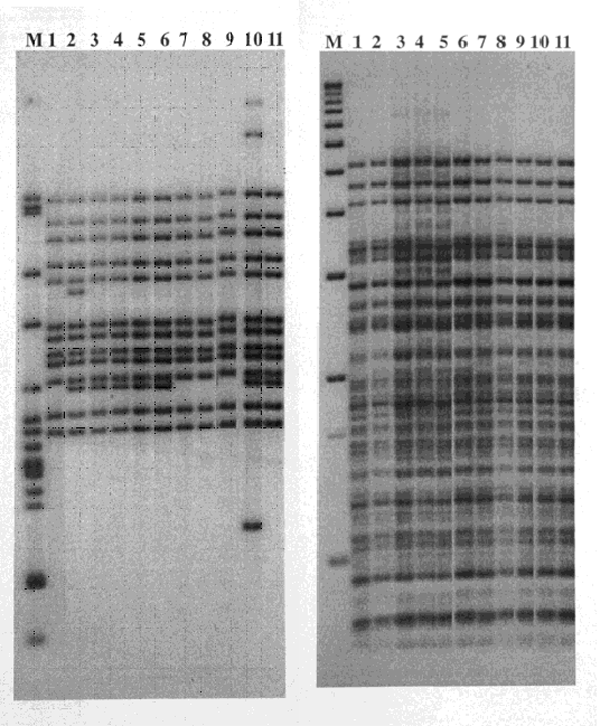
Restriction fragment length polymorphism patterns of Mycobacterium tuberculosis isolates from 11 patients residing in two geographically contiguous counties, Arkansas, 1992–1998. IS*6110* patterns are shown on the left and polymorphic GC-rich sequence on the right. Lane M shows M. tuberculosis strain H37Rv DNA marker (left) and 1-kb DNA ladder (right). Lane 1, isolate from patient 11; Lane 2, patient 13; Lanes 3–6, patients 4, 1, 3, and 2; Lanes 7–9, patients 10, 9, and 8; Lane 10, patient whose isolate differed by three bands and was not included in the study; and Lane 11, patient 5.

**Figure 2:**
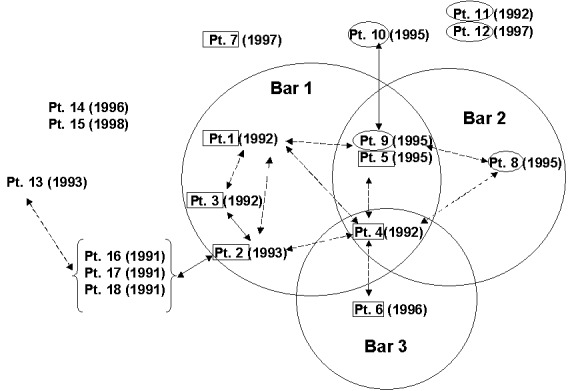
Epidemologic links among tuberculosis patients, Arkansas, 1992–1998. The circles represent the three public bars associated with the cluster of patients. Patient (Pt.) numbers enclosed with boxes and oval circles show patient isolates with patterns A and B, respectively. Parentheses show year of diagnosis. Solid black lines show epidemiologic links found during contact investigations. Dashed black lines show additional epidemiologic links discovered after DNA fingerprinting was done on the isolates and after standardized interviews were conducted with the clustered patients. Absence of lines means that no epidemiologic links were discovered for patients.

The 15-patient cluster included 7 patients whose isolates had an identical 13-band fingerprint pattern (pattern A) and 5 patients whose isolates had a DNA-fingerprint pattern that differed by one less hybridizing band (pattern B). The DNA fingerprint pattern of isolates from three other patients differed from one of the two primary patterns by a single band. Secondary typing showed that all 15 case-patients had an identical PGRS pattern (Figure 1). Before DNA fingerprinting, routine contact investigations had established epidemiologic links for only four patients. After this cluster was discovered, epidemiologic investigations demonstrated that 12 of the patients resided in the two geographically contiguous counties, and 10 additional epidemiologic links were established among patients in the 15-member cluster (Figure 2).

Of seven patients (patients 1–7) with pattern A isolates, we discovered epidemiologic links for six. These six patients were connected to three local bars (i.e., commercial drinking establishments). TB was diagnosed for four of the six patients in 1992 and 1993. We found links between four patients connected to bar 1, including the bar’s owner (patient 1), a bartender (patient 2), the bartender’s husband (patient 3), and a patron (patient 4). Patient 4 also frequented bars 2 and 3. TB was diagnosed in 1996 for patient 5 and patient 6, who frequented bars 2 and 3, respectively. Patients 5 and 6 were in contact in these bars with patient 4 when he was infectious (Figure 2). Patient 7 was not linked to any of these bars; we found no epidemiologic links for him.

We found epidemiologic links for three of five patients who had pattern B isolates. TB was diagnosed in 1995 for the three patients; two (patients 8 and 9) frequented bar 2, and the third (patient 10) was a granddaughter of patient 9. We could not find epidemiologic links for two patients who had pattern B isolates. Patient 11 was a nurse at the local hospital; no TB patients had been admitted to the hospital where she worked, and she did not remember caring for any TB patients. Patient 12 did not reside in the same city or county. Both patients 11 and 12 denied frequenting any of the bars (Figure 2).

An indirect epidemiologic link was found for one (patient 13) of three patients (patients 13–15). Patient 13’s isolate showed a DNA fingerprint pattern that differed from patterns A and B by a single band. Patient 13 was linked indirectly to patient 2 (bar 1) through acquaintances with patient 2’s family members (patients 16–18); TB was diagnosed for these patients in 1991. DNA fingerprinting was not conducted for isolates from patients 16, 17, and 18 because they had TB before the beginning of the study (Figure 1). No epidemiologic links were discovered for patients 14 and 15.

## Conclusions

Several factors are known to account for changes in RFLP patterns, including single nucleotide mutations that create a new restriction site or lead to loss of a preexisting site. Insertions, duplications, inversions, and deletions can cause changes in restriction fragments. In IS*6110* RFLP patterns, changes can also be accounted for by transposition of the insertion sequence itself. Although the IS*6110* RFLP pattern is sufficiently stable to enable us to make inferences about the linking of patients in a transmission chain, we have observed minor changes in the pattern (*15*).

DNA fingerprint clusters with similar but not identical IS*6110*-DNA fingerprint patterns should be investigated for epidemiologic links. In this investigation, we used secondary typing with PGRS and spoligotyping (data not reported) to show the isolates as indistinguishable. Previous studies demonstrate that the biological processes measured by PGRS typing or spoligotyping progress more slowly than those of transposable elements like IS*6110* (*11*). The molecular epidemiologic data indicate that the cases reported were caused by a strain of *M. tuberculosis* with the IS*6110* RFLP pattern that diverged only recently.

During the period 1996–2000, 719 TB cases were confirmed by culture in Arkansas; 707 were genotyped by using IS*6110*. Of cases with isolates having more than five copies of IS*6110*, 234 were in 59 clusters ranging from 2–16 cases. Nineteen of these clusters, including 101 cases, included at least one isolate that was similar but not identical to other isolates in the cluster. In a preliminary study, we discovered epidemiologic links among 15% of patients with IS*6110* RFLP patterns similar to those in clusters. During the same study period, we found epidemiologic links among 32% of Arkansas patients whose isolates shared an identical IS*6110* RFLP pattern.

In this cluster investigation, we discovered additional epidemiologic links among 10 (66%) of 15 patients that were missed during routine contact investigations. In addition, the investigation helped us link eight (53%) of the patients in the cluster to three local bars as common sites of TB transmission (*16*,*17*). Our investigation highlighted an extensive social network with multiple epidemiologic links (*18*).
